# Receiver response to high-intensity courtship differs with courter status in spotted bowerbirds *Ptilonorhynchus maculatus*

**DOI:** 10.1098/rsos.232015

**Published:** 2024-10-23

**Authors:** Giovanni Spezie, Dan C. Mann, Job Knoester, Thomas MacGillavry, Leonida Fusani

**Affiliations:** ^1^ Konrad Lorenz Institute of Ethology, University of Veterinary Medicine, Vienna, Austria; ^2^ Acoustics Research Institute, Austrian Academy of Sciences, Vienna, Austria; ^3^ Department of Behavioural and Cognitive Biology, University of Vienna, Vienna, Austria

**Keywords:** signal receiver, courtship, machine learning, startle response, visual display

## Abstract

Understanding sexual communication requires assessing the behaviour of both the sender and the receiver. Receiver responses to sexual displays carry relevant information, but such signals or cues may be subtle and therefore technically challenging to investigate. Here, we focus on receiver body movements in response to high-intensity courtship in spotted bowerbirds (*Ptilonorhynchus maculatus*). Male bowerbirds perform a vigorous courtship choreography on dedicated display structures—bowers. Bower owners tolerate other non-territorial males at their bowers, yet the courtship displays of these so-called ‘subordinate’ males rarely result in successful copulations. Males that display at high intensity are preferred by females in this species, yet excessively aggressive displays may be threatening, hence scaring prospective mates away. In this study, we hypothesized that bower owners are better able to exhibit high-intensity movements without startling their audience compared with subordinate males. To address this question, we used a combination of behavioural coding and AI-based tracking of body movements, which allows precise spatial and temporal resolution for the study of subtle behavioural responses. Contrary to our predictions, we found that bower owners evoked stronger startle responses than subordinate males. We discuss these unexpected results and suggest further experimental approaches for future investigations.

## 1. Introduction

Courtship displays often involve complex interactions between signallers and receivers. Even in species without mutual displays between the sexes [1,2], receiver behaviour can convey relevant information that may affect the pace and outcome of sexual interactions. For example, female cues have been shown to influence the timing of male courtship in whitethroats *Sylvia communis* [3] and substrate use by courting males in wolf spiders *Schizocosa rovneri* [4,5]. In particular, specific behaviours from females can signal receptivity and solicit mating [6,7] thus allowing courters to better coordinate copulation attempts [8]. Conversely, other distress cues such as startling may communicate discomfort towards excessively vigorous or persistent courtship. Male satin bowerbirds *Ptilonorhynchus violaceus*, for example, have been shown to adjust display intensity to these behavioural cues of discomfort from females [9–11]. Receiver responses can also produce long-term effects on signal production in courters, for instance, by affecting the developmental trajectory of courtship songs in brown cowbirds *Molothrus ater* [12] and postural displays in Amarillo fish *Girardinichthys multiradiatus* [13]. Thus, to fully understand the function and variation of sexual signals, it is essential to evaluate not only the sender’s behaviour but also how receivers respond to those signals [14].

Despite the importance of assessing receiver responses during courtship, such behavioural cues can be challenging and/or time-consuming to quantify by humans. While sexual displays are often relatively stereotypical and conspicuous, receiver responses may be subtle or difficult to assign to discrete categories, and these differences may in part explain why studies of courtship interactions have been predominantly biased towards courters [15]. Traditional methodologies such as behavioural coding may be inappropriate to quantify subtle movements in receivers; therefore, their responses have been typically recorded in terms of mere choice or preference (but see [16,17]). More recent technological developments have greatly expanded the potential to investigate sexual interactions from the perspective of receivers. For example, gaze-tracking on freely moving peahens (*Pavo cristatus*) revealed how females direct their attention to male courtship signals [18]. The emerging use of robotic animals to manipulate both sender and receiver behaviour has shed novel light on the interactive aspects of courtship displays [3,19–21]. Furthermore, motion capture and AI-based quantification of animal movements—which include object tracking and pose estimation—now allow precise and efficient tracking of body movements [22–26]. These methodologies hold great potential to quantify receiver responses with higher spatial and temporal resolution. Yet, in the context of courtship signalling, AI-based technologies have been more often deployed to study motor displays of courters, and controlled laboratory conditions remain the privileged setting for these analytical approaches [17]. Field conditions introduce several sources of noise that may hamper reliable and repeatable AI-based tracking of movements. For instance, the possibility of tracking body parts or even a whole animal across subsequent frames may be severely affected by occlusion by vegetation and background variability, for example due to changes in lighting and/or weather conditions, time of the day or number of animals present in the field of view [26]. Further technical requirements (simultaneous multi-device recordings, hardware synchronization and calibration) make it particularly challenging to quantify movement in more than two dimensions outside of the laboratory [26–27,28].

**Figure 1.**
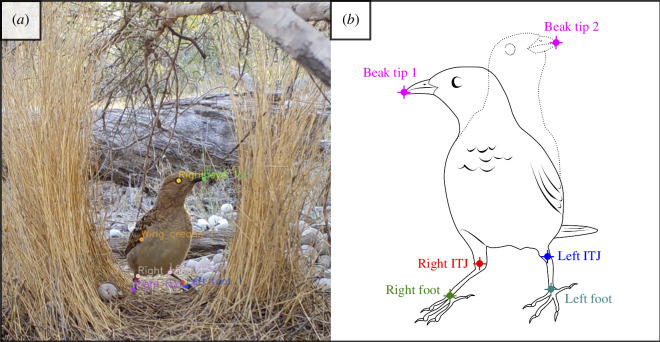
Automatic detection of keypoints on the bird’s body. (*a*) Screenshot of example output video showing a bird inside the bower walls, with automatically detected keypoints marked with individual labels. Only a subset of the keypoints shown in the picture was used for the analyses presented in this study. (*b*) Graphical representation of the keypoints of interest for this study. The dashed silhouette of the bird indicates the change in its body position between two subsequent frames (‘Beak tip 1’ and ‘Beak tip 2’). The Euclidean distance between beak coordinates in subsequent frames is used to calculate the variable ‘Relative displacement’ (RD) (see text). ITJ, inter-tarsal joint.

Here, we present a case study using two-dimensional tracking of receiver movements during courtship interactions in the wild. We investigated how receivers respond to high-intensity courtship displays in spotted bowerbirds (*Ptilonorhynchus maculatus*). Spotted bowerbirds exhibit particularly vigorous movements as part of their courtship routine [29], which includes harsh calls co-opted from aggressive displays [30,31] and violent body shudders [32,33]. During the breeding season, females visit display arenas and watch these elaborate audiovisual courtship choreographies after positioning themselves inside a bower (figure 1*a*)—a structure that is built and decorated by a resident male. Males that display at high intensity were shown to be preferred by females in this species [32], yet excessively aggressive displays can disrupt sexual interactions when vigorous body movements are threatening to receivers ([34]; see also [9,11]). In such cases, receivers interrupt courtship by hopping outside of the bower, away from the courting male (referred to here as ‘bower exits’), or exhibit startle responses, namely rapid reflexive body movements inside the bower [11].

Bower owners commonly tolerate the presence of other non-territorial males at their bowers. These males with subordinate status regularly visit established bowers and form stable male–male partnership with resident males [34,35]. During their visits, subordinates are displayed to by the owner, engage in bower building and perform courtship routines alone or to other male or female visitors ([34,35]; see also [36,37]). However, courtship displays by subordinate males are rarely successful and these males are only able to mate sporadically via sneaky copulations [38]. Several hypotheses have been put forward to explain the presence of subordinate males at bower sites. One possibility is that these males are tolerated because they contribute to enhancing the visual display of bower owners. A previous study, however, showed that higher subordinate attendance did not correlate with overall bower quality and decoration number but may instead contribute to bower defence and increase the conspicuousness of display sites to females [34]. Alternatively, as bower sites are limited and stable over time [39], subordinate males increase their chances of inheriting a bower when the owner dies [40]. Moreover, these juvenile males may gain additional delayed benefits from attending established bowers, such as learning the skills required for successful sexual signalling (bower-building and courtship) before they gain ownership of a display site (the ‘apprenticeship hypothesis’) [35]. For example, as overly vigorous movements have been shown to disrupt courtship by startling receivers [9,11], practising at adults’ bowers may allow subordinate males to refine their ability to minimize courtship interruptions and mitigate startle responses from their audience, though this latter hypothesis remains to be tested. Quantifying receiver responses to courtship displays from males with different bower ownership status may therefore provide valuable insights into the apprenticeship hypothesis.

Here, we hypothesized that bower owners are better able to deploy highly attractive—but potentially distressful—moves without startling receivers. To test this hypothesis, we assessed receiver body movements in response to courtship displays of males with different bower ownership status. We first used behavioural coding to compare the probability of bower exit events during courtship displays from bower owners and subordinate males. Then, we used a commercially available software to track more subtle movements by receivers inside the bower (startle responses) using AI-aided technology. We developed an analysis pipeline from the resulting *x* and *y* coordinates in order to track body movements in two dimensions and quantify startle responses in receivers. We predicted that receivers will be less distressed (i.e. lower probability of bower exits and weaker startle responses) when observing courtship displays of bower owners than those of subordinate males.

## 2. Material and methods

### 2.1. Study subjects and data collection

This study was conducted on a population of wild spotted bowerbirds at Taunton National Park (Scientific), Queensland (23.54989° S; 149.24088° E). We collected data during two breeding seasons in 2018 and 2019 (August to December). A total of *n* = 74 birds were caught at bowers using mist-nets and marked with individual combinations of five colour-bands and one metal band provided by the Australian Bird and Bat Banding Scheme (ABBBS). We used motion-activated camera traps (Browning Recon Force Advantage HD, 2018) to video-record activity at 13 bowers in both breeding seasons. Cameras were mounted on tripods or attached to a nearby support (distance from bower, mean ± s.d.; 2018: 120 ± 43.20 cm; 2019: 177 ± 46.67 cm). The frame rate for video recording was set to 30 frames per second. Bower ownership status of male birds (bower owner or subordinate male) was determined based on the size of the pink nuchal crest [41] and three behavioural features (bower attendance, maintenance and display rates at bowers), all of which were shown to significantly predict ownership status in previous studies [34]. Since spotted bowerbirds are sexually monomorphic [41], we took blood samples (*n* = 47) to confirm behavioural sex assignment via genetic sexing. In line with previous studies on the same population [41], our catch ratio was highly skewed towards males (42 males : 5 females), which resulted in our limited ability to obtain video footage of female visitors of known identity (see below). In addition, we could not precisely determine the age of marked individuals, as spotted bowerbirds older than 2 years show sexually mature plumage [42]. Among the male courters considered in the present study (bower owners: *n* = 12 individuals; subordinate males: *n* = 10 individuals), only one subordinate individual was identified as being younger than 2 years based on plumage and morphology [42].

### 2.2. Video selection and categorization of display elements in courters

A total of *n* = 956 courtship bouts were selected for behavioural coding (*n* = 22 male courters; mean ± s.d. = 43.45 ± 16.93 courtship bouts per courter). Courtship bouts were defined as strings of courtship display elements separated by intervals of less than 10 s (see [34]). We selected courtship bouts that included a banded courting male of known ownership status (bower owner or subordinate male) and a banded (*n* = 177 courtship bouts) or unbanded (*n* = 779 courtship bouts) visitor (hereafter, ‘receiver’) inside the bower. Unbanded receivers could not be sexed unambiguously through video recordings via morphological and/or behavioural traits (see [41]). These unmarked receivers may therefore include female bowerbirds that we were unable to catch and mark, as well as unbanded subordinate or other immature males that visited established bowers. Due to the limited availability of courtship bouts featuring banded female receivers (approx. 2% of courtship bouts), our sample did not allow us to conduct further formal analyses to investigate potential sex differences in receiver responses (see Discussion). Finally, we excluded from our analysis courtship bouts where female receivers exhibited the crouching posture that typically solicits copulations in this species and other bowerbirds [11,29]. Crouching females tolerate extreme levels of courtship intensity, and this posture was only observed during courtship bouts that preceded a copulation (G Spezie 2022, unpublished data).

In each courtship bout, all display elements performed by the courting male were manually coded using the software Loopy (http://loopb.io, Loopbio, GmbH, Austria), based on the repertoire of 19 discrete courtship elements described in Spezie & Fusani [34] (see electronic supplementary material, table S6). For each display bout, we annotated the time of occurrence of each element type, their duration and order within a sequence. By definition, ‘Event’ display elements had a duration of 1 frame, while ‘Duration’ display elements had a duration greater than 1 frame. More details about the coding procedure are described by Spezie & Fusani [34]. We additionally scored ‘bower exit’ events, namely each time receivers left the space between the bower walls during a courtship interaction (figure 1*a*). The display elements known as ‘body ripple’ and ‘mock attack’ were grouped into the category ‘high-intensity’ elements, as these moves typically occur at close proximity to the bower and significantly precede ‘bower exit’ events (see electronic supplementary material for details). All high-intensity elements were coded as ‘duration’ elements, with an average duration of 36.69 frames ± 35.92 frames (mean ± s.d., *n* = 2735). Other stationary display elements that typically occur further away from the bower—and did not significantly precede ‘bower exits’ in our sequence analysis (electronic supplementary material)—were categorized as ‘low-intensity’ [29,43].

### 2.3. Automatic detection of receiver movements

For the analysis of startle responses, we further curated the selected courtship bouts based on the position of receivers, in order to allow consistent tracking of body movements. As our methodology only allowed two-dimensional tracking of body movements, we dealt with depth by restricting our analysis to courtship bouts in which receivers were stationary on the *z* axis, namely the bird did not move longitudinally within the bower avenue (figures 1*a,b*; electronic supplementary material, video S1). For this analysis, our inclusion criteria for selection were that the receiver (i) was facing the camera throughout the courtship bout, with no occlusion by vegetation and (ii) did not exit the bower structure during a courtship bout. Thus, for the automatic tracking analysis of receiver movements, we only used courtship bouts that did not contain ‘bower exit’ events. A subset of 288 courtship bouts (30.16% of scored courtship bouts) met the above selection criteria and was used for automated analysis (bower owners: *n* = 9 individuals; subordinate males: *n* = 8 individuals; range 4–32 courtship bouts per male).

Selected courtship bouts were imported into the software Loopy (http://loopb.io, Loopbio GmbH, Vienna, Austria). We used machine learning to automatically track specific morphological features (hereafter ‘keypoints’) on receivers to examine their startle responses. The pose estimation function (‘pose detector’) that performs automatic keypoint identification on the bird’s body in the software Loopy uses the convolutional neural network ResNet50 [44] and is based on DeeperCut [45] with configurable stride. The pose detector returns *x* and *y* coordinates for each frame and keypoint (figure 1*b*; electronic supplementary material, video S1). In this study, the pose detector was not trained to track the movements of courters.

To train the pose detection algorithm (supervised machine learning), we first manually annotated keypoints on a subset of *n* = 55 courtship bouts. We selected these bouts from 13 bowers with different lighting conditions, distance of the camera from the bower and angle of the camera to the ground, in order to train the model on diverse sample data and increase its potential of generalization to novel videos. A total of 2167 frames of these 55 bouts (mean ± s.d. = 39.52 ± 3.80 frames per courtship bout) were annotated by G.S. and T.M., who manually assigned the position of five ‘keypoints’ corresponding to five distinct morphological features on the receiver’s body: beak tip, right and left foot, right and left inter-tarsal joints (ITJ) (figure 1*b*). These manual annotations were then used to train our keypoint detector with the following parameters: input network size (resolution of the images fed to the network) = 1344 × 756, stride (internal parameter of the convolutional neuronal network that describes a dimension reduction, i.e. simplification) = 4, and iterations (how often the data are shown to the network) = 150 000. Smaller stride values are more computationally demanding but increase the spatial resolution of the outputs. We then used the trained pose detector to run predictions on the full dataset (*n* = 277 822 frames, 288 courtship bouts; approximate duration of the analysis = 25 h) in Loopy (electronic supplementary material, video S1). Finally, we exported the keypoint coordinates for each separate courtship bout in a .csv format. These coordinates were used as input for further analysis in R [46] (see below).

### 2.4. Data cleaning and model validation

We conducted preliminary data-cleaning and validation steps before proceeding with the two-dimensional analysis of startle responses. To identify and remove incorrect detections (e.g. when a branch or bower decoration was detected as the beak of the bird), we calculated the average body size (in pixels) of a given bird in each courtship bout and then we filtered out the keypoints that deviated more than 0.5 body sizes from one frame to the next one (i.e. corresponding to an unrealistically high velocity of 1 body size per frame; *n* = 33, data loss: 0.01%). The remaining outliers that corresponded to realistic but very sudden changes in position of each given keypoint (i.e. data points that deviated between 0.25 and 0.5 body sizes; *n* = 86) were inspected visually (data loss: 0.003%).

We then verified the robustness of the automated (AI-based) annotation results. We validated the pose detector by comparing a random subset of the automatically annotated frames with respective manual annotations (by G.S. and T.M.) of the same frames, excluding those already used for training (see electronic supplementary material for the methodology and results of the validation procedure).

### 2.5. Quantification of startle responses within the bower

Using the *x* and *y* coordinates for all keypoints obtained from the software Loopy, we developed an analysis pipeline to quantify startle responses in receivers in R 3.6.2 [46]. For each frame, we first calculated the Euclidean distance (in pixels) between the position of the beak tip in that frame and its position in the previous frame (beak displacement; numerator of equation 2.1). We then divided this value by the bird’s average body height within each courtship bout (denominator of equation 2.1). Body height was calculated in each frame by subtracting the *y* coordinate of the beak and the *y* coordinate of the ITJ (figure 1*b*), then averaged across frames in the same courtship bout. We used ITJ because the model was less accurate in predicting feet positions (see electronic supplementary material).

This value of beak displacement relative to average body height, which we refer to here as ‘relative displacement’ (RD), is a measure of the amplitude of the bird’s head movements per frame in % of body height.


(2.1) $$RD=\;\frac{\sqrt{{\left(x_{Beak\;tip2}-x_{Beak\;tip1}\right)}^{2}+{\left(y_{Beak\;tip2}-y_{Beak\;tip1}\right)}^{2}}}{mean\left(y_{Beak\;tip}-y_{ITJ}\right)}.$$

**Figure 2.**
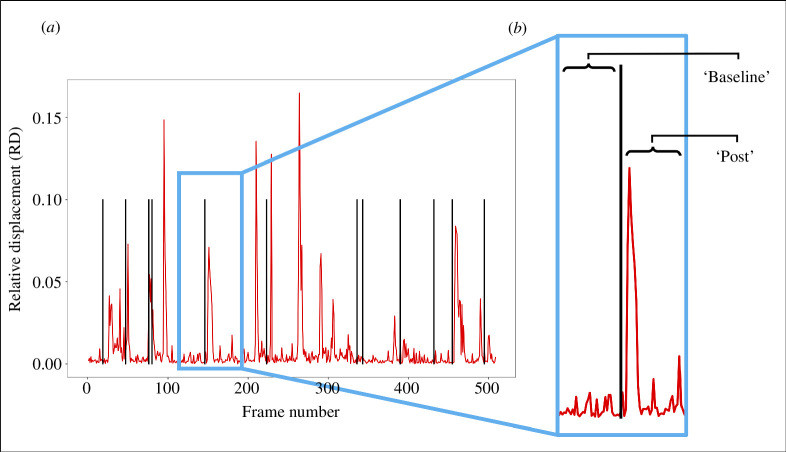
(*a*) Visual representation of the variable ‘relative displacement’ (red line) as a function of frame number in one example video of the duration of approximately 500 frames. A steep increase in relative displacement appears as a spike on the plot and indicates a startle response. Vertical black lines show the occurrence of display elements by the courting male. The position of the vertical line indicates the start frame of each behaviour. (*b*) Example of a window (light blue box) of a set number of frames that was used to isolate relative displacement values before (baseline) and after (post) the occurrence of display elements; the frame of occurrence was included in the calculation of both ‘baseline’ and ‘post’ segments.

For instance, a value of RD = 0.15 reflects a head movement that equals 15% of the bird’s body size (figure 2*a*). This value therefore quantifies rapid startle responses and, when averaged across frames, the overall restlessness of a bird in specific courtship segments.

Our final dataset therefore combined frame-by-frame information about the occurrence of display elements performed by the courting males (behavioural coding) and values of relative displacement in receivers (automated annotations; figure 2*a*). To investigate the effect of male courtship movements on receiver’s behaviour (startle responses), we isolated the values of relative displacement before and after the occurrence—namely, the start—of any given display element. We did so by selecting a window of a set number of contiguous frames around each display element (figure 2*b*). We calculated the difference between the *mean* relative displacement after any given display element (post) and the *mean* relative displacement before (baseline), and we referred to this measure as ‘Δ_mean_’.


$$\Delta_{mean}=mean\left(RD_{post}\right)-mean\left(RD_{baseline}\right).$$

Subtracting the baseline from the average relative displacement after a courtship movement removes the noise generated by the automatic annotation process, as well as controls for potential inter-individual differences in baseline restlessness in receivers. As an alternative method, we calculated Δ_max_ values by subtracting the maximum value of relative displacement in the ‘post’ and ‘baseline’ frames (see below). For both methods, the resulting metric ‘Δ’ quantifies the magnitude of the startle response produced by a display element on the receiver (figure 2*b*). Positive Δ values indicate an increase in mean or maximum relative displacement after a display element (i.e. a startle response), while negative Δ values indicate a decrease in mean or maximum relative displacement after a display element.

### 2.6. Statistical analyses

#### 2.6.1. ‘Bower exit’ events after high-intensity display elements

To study whether subordinate males are more likely to cause a bower exit than bower owners when performing a high-intensity element, we counted the number of high-intensity elements that were followed by a bower exit in both groups. We modelled this binary response variable (0 = no bower exit, 1 = bower exit) with a binomial distribution using the function *glmer* of the package *lme4* [47] and included bower ownership status as a predictor. We then repeated the same analysis only on the display element with the highest overall probability of bower exits (mock attack). Because we had repeated observations per subject and date, we included these two variables as random intercept effects into our model. A null model lacking the test predictor was compared with the full model using a likelihood ratio test and the R function *anova* [48].

#### 2.6.2. Effect of courtship behaviours on startle responses

To investigate the magnitude of startle responses evoked by subordinate males and bower owners, we calculated Δ values (see §2.5) after high-intensity display elements for bower owners (*n* = 9) and subordinate males (*n* = 8) separately. The number of video recordings and high-intensity behaviours analysed for each male varied considerably (range 4–32 and 45–790, respectively) thus our test statistics (Δ) were calculated as the average of the individual averages for both groups of males, to ensure that our results may not be driven by one or more specific individuals with larger samples sizes.

**Figure 3.**
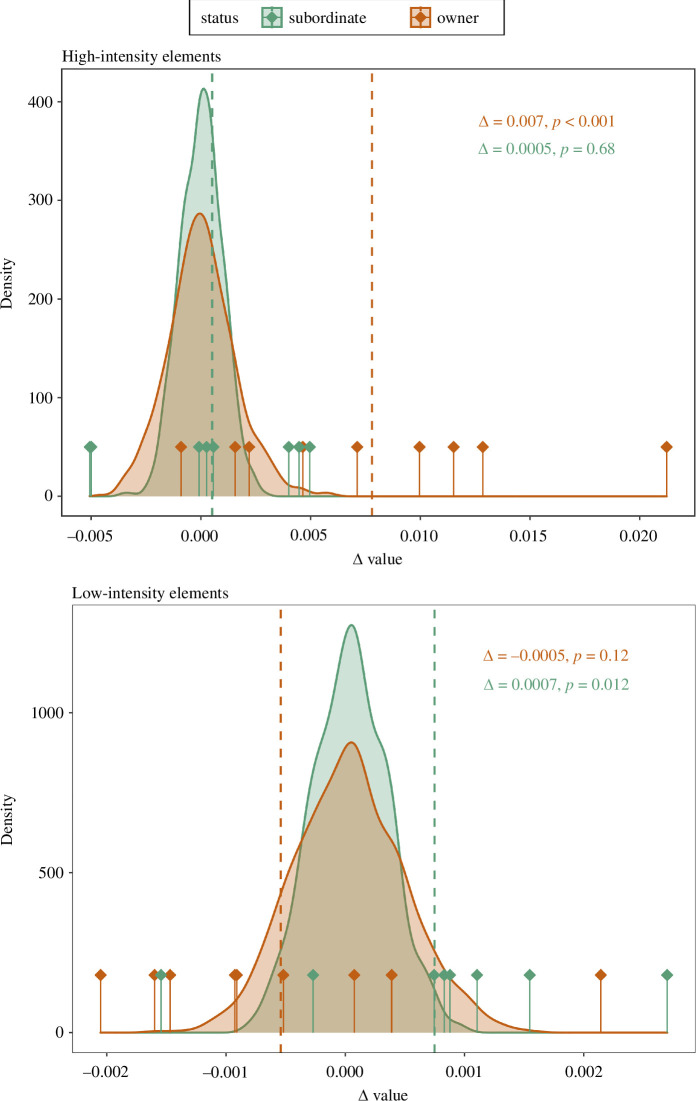
Density plots for bower owners (orange) and subordinate males (green) depicting the sampling distributions of 1000 Δ values calculated by bootstrapping random frames (window size = 10 frames) in the original dataset, compared with the observed mean Δ values (vertical dashed lines) after high-intensity (top) and low-intensity (bottom) display elements. Vertical solid lines with diamonds indicate the individual means. The mean Δ value for high-intensity elements in bower owners (dashed orange line) falls outside the 95% of the sampling distribution (*p* < 0.001).

**Figure 4.**
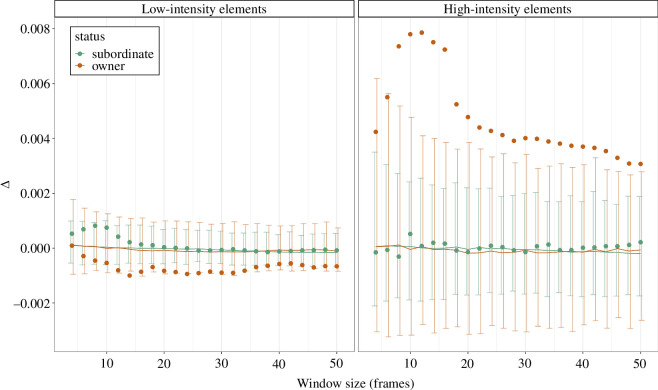
Results of the analysis of Δ values calculated using different window sizes (4–50) in subordinate males (green) and bower owners (orange). Results are shown separately for low-intensity elements (left panel) and high-intensity elements (right panel). Error bars depict for each window size the 2.5 and 97.5% limits of the distribution of Δ values calculated from randomly sampled frames. Observed mean Δ values (filled points) that fall outside of the 95% of the random distribution significantly differ from random. Solid lines connect the mean values of the random distributions.

Because little is known about the reaction time of bowerbirds, we decided against using a single window size (figure 2*b*). Prior research has shown that startle reaction times to light and sound stimuli in starlings (*Sturnus vulgaris*) range between 50 and 100 ms in laboratory conditions [49]. We therefore expected to detect startle responses at least within that range after the start of a display element. Yet, it is possible that reaction times and startle responses may vary depending on the identity or age of the receiver, and may therefore only be captured when looking at multiple window sizes, that is, after including additional frames in the ‘post’ segment of window size (figure 2*b*). We started our analysis with a ‘post’ size of *n* = 4 frames (approx. 133 ms), and then tested additional ‘post’ sizes of up to *n* = 50, recalculating estimates and *p* values at every step (see below), but keeping the baseline constant throughout, that is, equal to 10 frames. In order to better illustrate the details of our methodology, results are first shown for a window size equal to baseline size (*n* = 10 frames) (figure 3) and then for all window sizes combined (figure 4).

For each window size and status category, *p* values were calculated by bootstrapping random frames in the original dataset and comparing the observed Δ values with the randomly sampled distributions. This procedure acted as null hypothesis, as it allowed us to test whether the magnitude of startle responses after high-intensity elements was significantly larger than receiver responses during any random time in a courtship bout. In more detail, we sampled random frames from the full dataset of automatic annotation results (figure 2*a*) and recalculated Δ values for those randomly sampled frames. The mean was computed by randomly sampling in the dataset of each individual the same number of random frames as the number of high-intensity elements, and then this mean was averaged for bower owners and subordinate males. We repeated this operation 1000 times to obtain a sampling distribution for each group. In other words, the number of randomly sampled frames was equal to the number of frames used to calculate the test statistic of the observed data, that is, occurrences of high-intensity display elements for each group. The significance threshold for alpha values was set to 0.05.

As a control, we repeated the same analysis using Δ values for low-intensity elements. We predicted in particular that startle responses after low-intensity elements would not significantly differ in magnitude from Δ values calculated at random moments during courtship, as low-intensity elements—by definition—are not associated with elevated threat levels.

For both display element categories (‘high-intensity’ and ‘low-intensity’), we checked for possible influential observations and investigated the stability of our results by removing one male at a time and recalculating estimates and *p* values at every step. Furthermore, we repeated the entire analyses after varying the size (5, 10, 15 and 20 frames) of the baseline segment (figure 2*b*). The aim of this robustness check was to explore whether varying baseline size may yield different results than those obtained with a baseline size of 10 frames (see above). For example, accounting for larger baseline size may incorporate more noise into the analysis and consequently lead to overall lower Δ values.

## 3. Results

### 3.1. ‘Bower exit’ events after high-intensity display elements

The full models for high-intensity elements combined, as well as those for body ripples and mock attacks separately, did not fit significantly better than the respective null models without the predictor variable ‘bower ownership status’ (*χ*^2^ = 1.28, d.f. = 1, *p* = 0.25; *χ*^2^ = 0.87, d.f. = 1, *p* = 0.35, respectively). Thus, we found no evidence to suggest that bower ownership status has an effect on the probability of bower exits (electronic supplementary material, figure S1).

### 3.2. Effect of high- and low-intensity display elements on receivers’ movements

We first set window size to *n* = 10 frames and calculated mean Δ values for subordinate males and bower owners after high-intensity elements. The mean Δ value for subordinate males was within the 95% of the distribution of Δ values calculated at random frames during a courtship interaction (Δ = 0.0005, *p* = 0.69) (figure 3), suggesting that receivers did not respond more strongly to high-intensity elements of subordinate males than any random time during courtship. The mean Δ value for bower owners was larger than that of subordinate males and lay outside the 95% of the random distribution (Δ = 0.0078, *p* < 0.001). These results do not vary after removing possible influential observations (figure 3). For both subordinate males and bower owners, Δ values calculated using the alternative method (difference between *maximum* value of RD in the ‘post’ window and in the ‘baseline’, see above) yield comparable results to those obtained with subtracting *mean* values of RD, thus only the results of the latter are shown here.

These results are consistent and repeatable across different window sizes. The same effect of high-intensity elements persists when increasing window size up to 50 frames in bower owners (with fixed baseline size), and subordinate males do not evoke stronger startle responses than random chance even after allowing window size to increase (figure 4 and electronic supplementary material, table S4). In more detail, the significant increase in Δ values in bower owners appears to be particularly pronounced within a window size of about 20 frames (figure 4). Electronic supplementary material, figures S10 and S11 show the results at individual level. Overall, these results suggest that high-intensity elements evoke startle responses in receivers—that is, significantly stronger than random—only when performed by bower owners.

After low-intensity display elements, mean Δ values fell inside the 95% of the random distribution in bower owners (Δ = −0.0005, *p* = 0.12) but outside of the distribution in subordinate males (Δ = 0.0007, *p* = 0.012) (figure 3). These results suggest that subordinate males evoked startle responses in receivers after low-intensity displays. However, the results for low-intensity elements varied depending on the window size we set (figure 4, electronic supplementary material, table S5). In subordinate males, the effect of low-intensity elements on startle responses is limited to three window sizes (figure 4). In bower owners, the analysis on larger window sizes revealed that low-intensity elements evoked significantly below-average movements in receivers (electronic supplementary material, table S4), suggesting a freezing response.

Finally, the results for subordinate males were partly unstable when different baseline sizes were used. With larger baseline values (baseline size = 15 and baseline size = 20) for high-intensity display elements, the Δ values for subordinate males were significantly lower than the mean of the random distribution for all values of window size (electronic supplementary material, figures S13 and S14). Thus, high-intensity elements in subordinate males seem to be followed by a decrease in receiver’s movements when accounting for larger baseline size.

## 4. Discussion

This study confirms the reliability of AI-based quantification of body movements for behavioural research on sexual communication in a wild setting. The results of our validation show that the data generated by the automated tracking were highly correlated with manual annotations by human observers. Prior studies primarily relied on human observers to quantify receiver responses to courtship signals, though behavioural coding presents several limitations for precisely capturing body movements while limiting the risk of observer bias (see [50]). AI-based tracking allowed us to focus on specific aspects of sexual signalling that would be otherwise challenging (or extremely time consuming) to quantify, particularly outside of the laboratory.

The aim of this study was to assess receiver movements in response to live displays in a species with vigorous courtship. We hypothesized that the probability of bower exits and the magnitude of startle responses in receivers may vary depending on the status of the courting male. In particular, we predicted that bower owners would cause receiver to startle less in response to high-intensity courtship. The first part of our analysis showed that subordinate males were not more likely to cause bower exits than bower owners. Bower exit events appear to be a common response to extreme levels of courtship intensity in this species, and these responses were evoked by all males in our study, irrespective of their bower ownership status. These responses may be analogous to a flight reflex to threatening stimuli when males charge the bower entrance during extremely vigorous display elements and slam their body against the bower walls (mock attack) [32,34]. Also, bower exits may play a role in preventing forced or unsolicited copulations before mating decisions are made by receptive females [30]. Because our analysis excluded receivers with the typical copulatory crouching posture [11], most females in our dataset were plausibly not ready to mate and may have not tolerated physical proximity to approaching males, irrespective of their attractiveness. As such, behavioural coding of bower exit events may not be a suitable approach to capture variation in receiver responses to male courtship.

In contrast, the AI-based tracking of more subtle movements inside the bower provides a more complex picture. When focusing on startle responses, our results show that bower owners evoked startle responses in receivers after highly intense courtship, while subordinate males did not. The finding that high-intensity elements performed by bower owners—but not subordinate males—significantly startled receivers warrants further discussion. In a previous study, Patricelli *et al*. [9] demonstrated that startling rate is inversely related to male courtship success in satin bowerbirds. However, this latter study focused only on the courtship behaviour of sexually mature bower owners, while our analysis targeted both bower owners and subordinate males. Thus, a possible interpretation of our results is that stronger receiver responses to motor courtship may overall reflect the level of stimulation, responsiveness and engagement that courters are able to produce in their audience. Previous work has shown that mate choice selects for elevated courtship vigour in this species, and high-intensity courtship components positively correlate with mating success in spotted bowerbirds [32,33]. Receiver startling may be associated with sudden changes in intensity within temporally dynamic displays, which might have a positive effect on overall courtship success when deployed with appropriate timing [51]. Subordinate males may, therefore, be unable or not motivated to display at performance maxima, which would result in overall lower responsiveness from receivers. In line with Patricelli *et al*. [9], we also found variation within bower owners in the magnitude of receiver startling (figure 3), and future studies should clarify the link between receiver behaviour and mating success in this species, for example, by investigating how variation in receiver responses may ultimately influence mate choice. In addition, while our analysis only included visual signals, previous studies investigating display intensity in this species have also looked at variation in acoustic courtship components [30–32]. The harsh vocalizations used by males during courtship displays may indeed play a significant role in enhancing the intensity of visual display elements with a potential effect also on receiver responses. This possibility, however, remains to be explored, particularly after comparing males with different bower ownership status.

Furthermore, since we could not band and identify all individuals in our study population, we lack relevant information about unbanded receivers. Female satin bowerbirds have been shown to visit the same males repeatedly before mating [52,53], and female startling decreases after repeated courtships with the same males [10]. Also, prior studies in the same species suggest that mate searching tactics vary with female age [54], and that older females are less easily startled by intense displays [10,54]. Furthermore, sex-driven differences in the magnitude of startle responses should be further explored in future studies. We might expect that males would be less likely to exhibit startle responses than females, particularly because males should be less sensitive to highly intense courtship cues, whereas females need to be sensitive to cues to avoid forced copulation attempts [51]. Although both male and female receivers of known identity appeared to exhibit bower exits and startle responses during high-intensity displays, the potential effect of receiver sex and age on the magnitude of bower exits and startle responses was not further explored in the present study due to our limited knowledge about the demographics of our study population. Thus, it would be informative to account for the identity, age and reproductive status of female and male receivers in order to have a more complete understanding about the variation in receiver response to highly intense courtship. Finally, to further refine our design, one could incorporate information about bower architecture and decorations of each arena, which were shown to affect receiver startling in satin bowerbirds [55].

While our results point to a clear difference in the way receivers respond to high-intensity courtship from bower owners and subordinate males, we still lack an understanding of the mechanisms that may explain this variation. As stated above, subordinate males may display with overall lower intensity, for example, by displaying further away from the bower or by reducing the loudness of courtship calls, the rate of decoration tossing or of body shuddering [32]. Alternatively, males of different age and/or status may vary in their ability to deploy high-intensity elements at suitable moments during a courtship bout (see [56–58]). In support of this, we found that subordinate males significantly startled receivers after low-intensity elements, and the opposite effect was exerted by low-intensity elements in bower owners, which again suggests that immature birds employ certain display components with inappropriate timing. It should be noted, however, that this latter effect was weak and repeatable only with three values of window size; therefore, this latter result should be interpreted with caution.

The apprenticeship hypothesis posits that subordinate males attend established bowers to practice bower building as well as their motor displays [35]. Visiting adult males’ bowers during development may also allow subordinate males to learn how to correctly deploy vigorous display elements by practising with other birds. Developing the skills required for attractive and successful sexual signalling has been shown to be contingent upon experience and practice in a number of avian species [59]. For instance, in the lek-breeding swallow-tailed manakin (*Chiroxiphia caudata*), Schaedler *et al*. [58] found that juvenile males exhibit more stereotyped courtship choreographies than adult birds and suggest that younger birds may learn context-specific use of specific display components with practice and experience. Though we did not have accurate information about the age of subordinate males, morphological and behavioural features in this and other closely related species suggest that subordinate males are indeed sexually immature [35–37]. Future studies should collect long-term data on the same individuals in order to investigate the temporal changes of courtship motor components in juvenile males. For instance, Soma *et al*. [57] investigated the longitudinal changes in postural components of courtship in Java sparrows (*Lonchura oryzivora*), and found a gradual increase in the coordination of audiovisual courtship components during development. Longitudinal data and developmental information would provide more conclusive evidence to support the hypothesis that motor competence is gained gradually and via an extended period of protracted motor training at established bowers. Moreover, experimental manipulation of social interactions during development would shed more light on the role of individual versus social learning in courtship development.

A second explanation is that receivers may be able to infer courter status from morphological traits or via other social behaviours and hence modify their behavioural responses. Prior research showed that subordinate males have smaller nuchal crests than bower owners [41] and rarely exhibit male-specific behaviours (courtship, maintaining) in the presence of bower owners [34]. Thus, receivers—and male receivers in particular—may perceive lower threat levels while attending the displays of subordinate males than those of bower owners, independently of specific features of their courtship. To rule out this alternative scenario, the next step is to quantify the movements of courters along with those of receivers. AI-based quantification could be deployed in future studies to precisely quantify the body postures and trajectories of courtship movements in courters in order to examine which fine-scale characteristics are more likely to differ with bower ownership status or during development. In addition, a similar approach could be used to investigate whether males adjust their courtship behaviour in response to receiver startling in this species. For instance, male satin bowerbirds (*Ptilonorhynchus violaceus*) were shown to adjust motor performance based on audience reactions by modulating display intensity when receivers showed cues of distress ([9,11]; see also [32]). Thus, assessing whether male spotted bowerbirds use specific courtship elements depending on the behaviour of receivers would suggest a link between experience and responsiveness to female startling in this species. A growing body of empirical evidence suggests that courtship displays can often be regarded as dynamic interactions, where courter behaviour and receiver responses affect each other in a mutual fashion [14]. In particular, sexual traits that are indicative of courters’ abilities to process and integrate exogenous information may in turn reflect their developmental history and overall condition thus clarifying the link between cognitive skills and sexual selection ([60–64]; reviewed in [65]).

In conclusion, our results strongly suggest that assessing receiver behaviour is an important step for understanding the function and evolution of sexual signals [14,66]. Our methods show that automatic tracking of specific body parts via machine learning techniques (i.e. pose estimation) can be reliably implemented in a wild setting. Pose estimation and automated movement tracking are becoming an increasingly popular tool to investigate motor performance and gestural communication [23–26]. A growing number of studies also shows that mate choice often targets highly coordinated and rapid movements [67,68], some of which may not be perceived by human vision [69–71]. These techniques may therefore be more and more needed in the future to efficiently and accurately quantify complex and/or subtle movements that would otherwise require hundreds of hours of manual work. Finally, addressing questions about courtship motor performance in the wild can greatly expand the range of model systems to include more species where motor displays are difficult to study in a captive setting.

## Data Availability

Datasets and codes can be accessed as electronic supplementary material [72].
